# Exploring the Role of Learning Styles and Motivation in Medical Student Engagement and Academic Performance: A Mixed Methods Study

**DOI:** 10.5152/eurasianjmed.2025.25862

**Published:** 2025-09-03

**Authors:** Selçuk Akturan, Fatmanur Keski, Çağrı Berk Sağlam, Zeynep Büşra Sezer

**Affiliations:** Department of Medical Education, Karadeniz Technical University Faculty of Medicine, Trabzon, Türkiye

**Keywords:** Academic performance, learning styles, medical education, motivation, VARK model

## Abstract

**Background:**

Although numerous studies have examined the relationship between learning styles and factors such as gender, academic performance, and participation, no research has been found that comprehensively investigates the interplay between learning styles, motivation (intrinsic and extrinsic), and academic performance across diverse educational activities. This study aims to explore the influence of different learning styles and motivations (intrinsic and extrinsic) on medical students’ engagement and academic performance.

**Methods:**

Using a mixed-methods approach, the study combined quantitative data from 169 medical students with qualitative insights from focus groups and in-depth interviews with 27 participants. Quantitative data were analyzed using descriptive statistics and Chi-square tests, while qualitative data were thematically analyzed.

**Results:**

Findings revealed no significant direct relationship between learning styles and academic performance. However, intrinsic motivation was strongly associated with higher academic achievement. Female students showed a notable preference for visual and kinesthetic learning styles. Practical, hands-on educational activities, such as laboratory work and simulations, were linked to greater engagement, particularly for kinesthetic and visual learners. Students emphasized the importance of personalized feedback and diverse teaching methods in enhancing motivation.

**Conclusion:**

While learning styles alone do not predict academic performance, motivation, especially intrinsic motivation, plays a critical role in student achievement. The findings highlight the importance of using interactive and varied teaching methods that cater to different learning styles and foster motivation. Medical curricula should focus not only on accommodating diverse learning styles but also on strategies to enhance intrinsic motivation to improve academic outcomes.

## Introduction

The transition from high school to the first year of medical school, with its heavy curriculum, can be challenging for students. Medical students come from diverse backgrounds in terms of high school experience, culture, ethnicity, and preparation levels, representing a wide spectrum. This diversity also poses challenges for educators in designing and implementing effective educational programs. It is the responsibility of educators to address the diversity of learning styles among students and to develop appropriate learning approaches.^1^ Learning style refers to an individual’s preferred way of processing information, typically categorized as visual, auditory, reading/writing, or kinesthetic (VARK).^2^ Neil Fleming’s VARK model is one of the most commonly used tools to identify students’ specific learning styles. Fleming proposed a tool in 1987 that identifies 4 main types of learners: visual, auditory, reading/writing, and kinesthetic.^3^ In general terms, visual learners prefer watching videos, images, and diagrams; auditory learners learn by listening to lectures; reading/writing learners prefer reading texts and taking notes; and kinesthetic learners learn by touching and manipulating objects.^4^

Recent studies suggest a complex relationship between learning styles and learning motivation among medical students. While students may have distinct preferences for learning (e.g., visual, auditory, kinesthetic), research indicates that motivation often plays a more significant role in academic performance. A study by Husmann and O’Loughlin^5^ found that medical students with intrinsic motivation, regardless of their learning style, performed better academically.

Several studies have examined the relationship between learning styles and academic performance among students from different backgrounds.^6^^-^^8^ However, studies comparing high-achieving and low-achieving student groups have not found significant differences in learning styles. Moreover, no significant relationship between gender and learning styles has been demonstrated.^9^^,^^10^

These studies suggest that the dominance of certain learning styles among students may be related to their field of study, learning methods, learning experiences, curriculum content, and the intensity of the course content. Therefore, it is recommended that educators consider learning style differences when planning lessons and adopt a variety of teaching methods accordingly.^11^^,^^12^Although many studies in the literature have examined the relationship between learning styles and variables such as gender, academic performance, and participation, no study has investigated the relationship between learning styles, motivation, and academic performance across various educational activities from a holistic perspective. This study draws on Constructivist Learning Theory, emphasizing knowledge construction through experience and interaction^13^^,^^14^, and Kolb’s Experiential Learning Theory, which frames learning as a cyclical process involving experience and reflection.^15^

This study aims to explore the influence of different learning styles and motivations (intrinsic and extrinsic) on medical students’ engagement and academic performance. It seeks to understand how adaptability in learning styles can enhance academic performance within a constructivist learning environment and investigates the role of collaborative learning settings in aligning students’ learning preferences with their motivation and achievement. The following sub-questions will also be addressed:

1. How do medical students’ learning styles (visual, auditory, reading/writing, kinesthetic) influence their engagement and active participation in pre-clinical education activities, and how does this vary across different levels of intrinsic and extrinsic motivation?
2. What role does adaptability in learning styles play in enhancing academic performance among medical students in a constructivist learning environment, and how can this be fostered through curriculum design?
3. How do collaborative learning environments in medical education impact the alignment of students’ learning styles with their motivation and academic achievement?

## Materials and Methods

### Research Design

This study employs a mixed-methods design.

### Location and Time Frame of the Study

This study was conducted at Karadeniz Technical University School of Medicine between September 2022 and May 2024.

### Sample

The population of the study includes first-, second-, and third-year medical students. For the quantitative VARK questionnaire, a stratified sampling method was used, with at least 127 students selected at a 95% confidence level and 0.8 power effect. To account for sample loss, 10% more students will be included (a total of 141 students, with at least 47 students from each class). For qualitative interviews, 8 students were invited from each year (3 groups) who completed the questionnaire using convenience sampling. The invited students were selected to ensure at least 2 participants from each class. Since the researchers considered data saturation to have been reached, no additional focus group interviews were deemed necessary.

### The Research Phases

**Figure 1.**
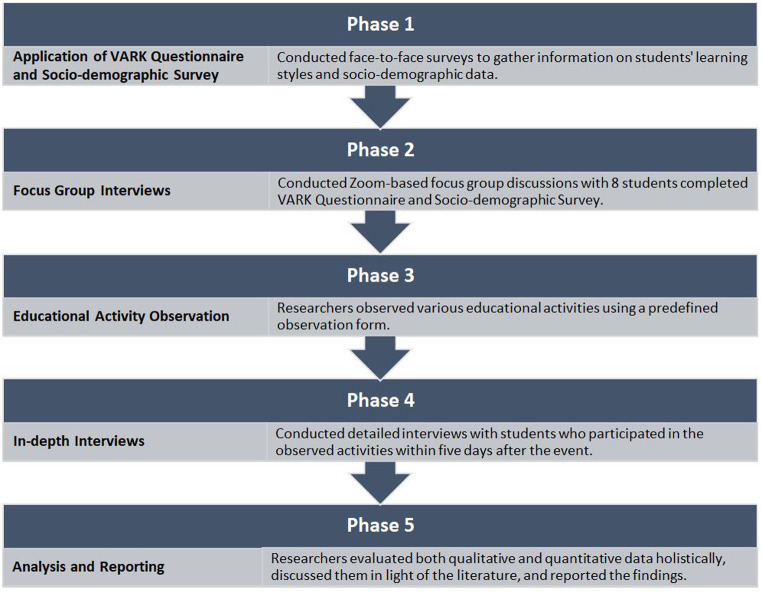
The research process outline.

This study was conducted in 5 phases, as shown in Figure 1.

### Data Collection Tools

Data collection tools are listed below:

1. Visual, auditory, reading/writing, or kinesthetic Questionnaire (Supplementary material- 1): Developed by Neil Fleming in 1987, the VARK Learning Styles Questionnaire identifies how individuals exchange information and their preferred sensory modalities for learning or teaching.^16^ The questionnaire presents 16 scenarios, each depicting a situation in which participants must choose how they would act. The dominant learning style that emerges from their responses indicates their learning style. The VARK questionnaire effectively measures visual, auditory, reading/writing, and kinesthetic/tactile styles.^17^ The Turkishversion of the VARK Learning Styles Questionnaire, validated by Leite et al, has a reliability coefficient of 0.76.^18^
2. Socio-demographic Information Form (Supplementary material- 2): This form, consisting of 5 questions, was applied alongside the VARK questionnaire.
3. Educational Activity Observation Form (Supplementary material- 3): Researchers attended educational activities that used different learning methods, with permission from instructors, and evaluated these activities using a form developed by the researchers. Before conducting the observations, a consensus meeting was held under the leadership of the principal investigator, with the participation of the other researchers who would conduct the observations. This meeting aimed to clarify key considerations related to the research objectives and questions, as well as how to interpret the items on the observation form. Particular attention was given to ensuring that the researchers, who are also the observers, did not observe the educational activities of the same class they were involved within the study. Additionally, when determining the educational activities to be observed, several factors were taken into account, including the use of various learning methods from different academic periods, activities led by different instructors, the availability of the researchers conducting the observations, and the approval of the instructor responsible for the selected educational activity. Researchers observed various learning methods until data saturation was achieved. Observation notes were converted into reports by the observers. The reports were subjected to qualitative content analysis.
4. Qualitative Interview.

Students’ perspectives have been uncovered using qualitative methods, specifically focus group discussions and in-depth interviews.

Focus group interview: Semi-structured interviews were conducted with students in focus group discussions based on the following questions:

- How do the methods used in educational activities affect your motivation?
- How do visual materials (e.g., videos, images, and diagrams) used in educational activities impact you?
- How do auditory materials affect you?
- How do written materials (lecture notes, writing) impact you?
- How do interactive materials (e.g., simulated patients, models) affect you?

The focus group interview was conducted in a suitable room at the Department of Medical Education, and an audio recording was made. Participants provided verbal consent before the recording. The focus group discussion lasted approximately 1.5 hours.

In-depth interview: Semi-structured in-depth interviews were conducted with students identified during the researcher’s observations. When selecting participants, an attempt was made to invite 1 person from among those who actively participated during the observation (asking questions, answering questions, expressing their opinions, etc.) and 1 person from among passive participants. The interviews lasted approximately 20 minutes, and the students were invited for the interview at the Department of Medical Education as soon as possible after the class (within a maximum of 2 days). Semi-structured in-depth interviews conducted with the students identified through researcher observations were based on the following questions:

- Could you share with us a situation or event during the lesson that had a positive or negative impact on you?
- Could you share your thoughts on how the educational activity you participated in affected your education?
- What changes in the educational activity would help you benefit more from it?

### Data Analyses

#### Quantitative Data Analysis

For the evaluation of quantitative data, the SPSS 25 program (IBM SPSS Corp.; Armonk, NY, USA) was used, and in addition to descriptive statistics, Pearson chi-square, *t*-tests, and variance analyses were conducted to reveal correlations and test the significance of differences.

#### Qualitative Data Analysis

For qualitative data, Creswell’s qualitative data analysis approach was adopted.^19^ The audio recordings were transcribed into written text by the researchers. Before starting the coding process of the research texts, the principal investigator, who is experienced and an expert in qualitative analysis, organized a practical course on the analysis of qualitative data for the other researchers. After the course, all data were coded by the researchers, and the codes were finalized through meetings held until a consensus was reached. Following the coding process, additional meetings were conducted by the researchers to identify sub-themes and themes. After determining the themes and sub-themes, the researchers were divided into 2 groups to re-examine the entire texts, testing the consistency and validity of the themes and sub-themes. Finally, the themes and sub-themes were interpreted and reported in conjunction with the current literature.

### Ethical Approval

This study was conducted in accordance with the ethical standards of the Declaration of Helsinki. Approval for the study was obtained from the Karadeniz Technical University Faculty of Medicine Clinical Research Ethics Committee (Approval no: 2022/15; Date: March 25, 2022).

## Results

The VARK Questionnaire and Socio-demographic Information Form were administered to 142 students. Among these, 8 volunteers participated in a focus group discussion. Each of the 3 academic years was represented by at least 2 participants among a total of 8 participants (Year 1: 2 participants, Year 2: 3 participants, Year 3: 3 participants). Observations were conducted in a total of 16 educational activities, with the breakdown of these activities by year listed below:

- Year 1: 2 clinical skills training sessions, 2 lectures, 1 laboratory skills training, and 1 problem-based learning session (a total of 6 educational activities),
- Year 2: 1 lecture, 1 multidisciplinary case-based session, 2 problem-based learning sessions, and 2 laboratory skills training sessions (a total of 6 educational activities),
- Year 3: 1 lecture, 2 multidisciplinary case-based sessions, and 1 laboratory skills training session (a total of 4 educational activities).

In-depth interviews were conducted with 27 students who volunteered from the observed educational activities. Of these, 8 were from Year 1, 11 from Year 2, and 8 from Year 3.

### The Analysis Results of the Visual, Auditory, Reading/Writing, or Kinesthetic Questionnaire and Socio-Demographic Information Form

The VARK questionnaire and the socio-demographic information form were administered to a total of 169 students, including 62 (43.7%) from Year 3, 54 (38.0%) from Year 2, and 53 (18.3%) from Year 1. There was no significant difference between the classes in terms of learning styles (*P* = .385). However, female students significantly preferred visual and kinesthetic learning styles (*P* = .014). There was no significant difference between the most preferred/enjoyed learning method and learning styles (*P* = .805). No significant correlation was found between receiving mentoring during undergraduate education and learning styles (*P* = .659). The frequency analysis results and relationships of the questionnaires are detailed in Tables 1 and 2.

### Content Analysis Results of Focus Group Interviews with Students

Based on the 3 focus group interviews with students, several key themes emerged regarding learning motivation, the role of practical education, and the use of teaching materials. As detailed in Table 3, students emphasized that teaching methods and instructor approaches significantly impact their motivation to participate in educational activities. Practical and interactive lessons, particularly those involving laboratory experiences and simulations, were highlighted as the most effective in sustaining student engagement. Additionally, visual and auditory materials, especially animations and simplified slides, were seen as critical in facilitating comprehension of complex theoretical concepts. Peer education also emerged as a preferred method for reinforcing learning, especially in hands-on environments. In contrast, monotonous, lecture-based sessions with slide-heavy presentations were identified as demotivating and less effective in promoting active participation.

### The Analysis Results of Observation Notes: Comparative Evaluation of Teaching Methods

The comparative analysis highlights the varying effects of different teaching methods on student engagement, attention, and overall learning outcomes. When appropriately selected based on the instructional goals and the student cohort, these methods can significantly influence educational effectiveness.

- Interaction Levels
- Most interactive methods: Problem-Based Learning (PBL) sessions and laboratory-based lessons demonstrated the highest levels of student engagement and educator-student interaction.
- Least interactive methods: Lecture hall classes and multidisciplinary sessions created more passive learning environments, resulting in lower levels of student participation.
- Attention Retention
- Highest retention: Laboratory lessons and PBL sessions were most effective at sustaining student attention, attributed to their practical, student-centered approaches.
- Lowest retention: Lecture hall classes and multidisciplinary sessions were associated with reduced attention spans, particularly in extended sessions with slide-heavy presentations that led to decreased student engagement.
- Participation in the Learning Process
- Highest participation: PBL sessions facilitated the greatest student involvement in active learning, with group work and scenario-based activities fostering critical independent thinking skills.
- Lowest participation: Lecture hall classes were predominantly lecture-driven, offering minimal opportunities for active student participation.
- Overall Efficiency
- Most efficient methods: Laboratory lessons provided a strong reinforcement of theoretical knowledge through practical application, while PBL sessions were effective in enhancing critical thinking skills.
- Least efficient method: Multidisciplinary sessions struggled to maintain student engagement during prolonged lectures and required supplementary interactive activities to enhance effectiveness.

The details about the comparison of educational methods are shared in Table 4.

### The Data Revealed from In-Depth Interviews with Students

The analysis results revealed the experiences of students based on different educational methods, physical conditions, and personal learning styles. The 6 main themes identified—effectiveness of educational methods, physical conditions, interaction with instructors, student motivation, personal learning styles, and learning challenges—along with their related sub-themes, highlight how students experience the learning process and what factors positively or negatively influence this process. The analysis revealed several important findings: students placed greater emphasis on practical education and individual study, experienced attention and motivation issues during theoretical lessons, and found the lack of physical resources and large class sizes to negatively impact the learning process. Additionally, students expressed a desire for more individual feedback and closer interaction with instructors (Table 5).

## Discussion

### The Impact of Different Learning Styles on Student Engagement and Academic Performance

This study explores the interaction between learning styles, motivation, and student engagement, with a particular focus on the VARK model as a tool for understanding learning preferences in medical education. Although previous research has widely applied the VARK model to categorize learning styles^5^^20^, the findings indicate that learning styles alone do not have a decisive impact on academic performance. Instead, motivation—particularly intrinsic motivation—emerges as a more significant factor influencing student performance.^21^

While aligning teaching methods with students’ learning preferences may increase engagement in the short term^22^, the findings suggest that this approach must be supplemented by strategies that foster motivation. This study also fills a gap in the literature by highlighting how learning styles can influence engagement, particularly in practical and interactive learning environments, such as laboratory sessions and simulations. Kinesthetic and visual learners showed greater engagement and success in these settings, corroborating previous research that underscores the benefits of hands-on learning for kinesthetic learners.^4^ Similarly, the use of visual aids has been shown to enhance comprehension for visual learners.^23^

### The Complex Relationship Between Motivation, Academic Performance, and Learning Styles

This study emphasizes that motivation, particularly intrinsic motivation, is more predictive of academic performance than learning styles alone. Students with higher levels of intrinsic motivation consistently demonstrated better academic outcomes, regardless of their learning preferences. This supports the findings of Iqbal et al,^24^ who also identified intrinsic motivation as a critical factor in student performance. Consequently, educational programs must go beyond catering to learning styles and instead focus on cultivating intrinsic motivation through interactive and student-centered teaching methods.

Interactive and practical lessons were found to significantly increase motivation, while monotonous, lecture-based approaches, particularly those relying heavily on slide presentations, had the opposite effect. These findings align with existing literature emphasizing the importance of varied teaching methods to maintain student interest and engagement.^12^^25^ Instructors play a crucial role in adapting their teaching strategies to match the diverse learning preferences of students, which in turn can foster higher motivation and engagement. This dynamic interaction between teaching strategies and motivation is essential for promoting academic performance.

### A Contribution to Mixed Methods Research

This study contributes to mixed-methods research by integrating both quantitative and qualitative approaches to gain a comprehensive understanding of learning styles, motivation, and academic engagement. The combination of statistical analysis and thematic evaluation provides a multidimensional perspective, bridging the gap between numerical trends and individual experiences. By utilizing mixed methods, this research not only confirms previous findings regarding motivation and engagement but also deepens the understanding of how learning preferences shape student experiences in medical education. These findings highlight the importance of using multiple methodological approaches to develop holistic educational strategies.

### Practical Implications for Medical Education

Our study highlights the importance of integrating diverse teaching methods into medical curricula to enhance both engagement and practical competencies. Practical, hands-on sessions, such as laboratory work and simulations, were particularly effective for kinesthetic and visual learners, underscoring the need for medical education programs to incorporate more interactive components. This shift towards a more practical and student-centered approach can enhance engagement and motivation, particularly in complex or dense subjects where student participation tends to wane.

Furthermore, the role of personalized feedback in fostering student engagement cannot be understated. Timely, individualized feedback has been shown to act as both a motivational tool and a reinforcement mechanism, particularly in student-centered learning environments.^26^ This finding aligns with current educational theories advocating for increased student-instructor interaction as a means to deepen learning and promote cognitive engagement.^27^ Medical educators should prioritize the integration of personalized feedback and mentorship to better prepare students for the complexities of clinical practice.

### Future Directions for Research and Educational Practice

Our findings suggest that future research should focus not only on the short-term effects of learning styles and motivation but also on their long-term impact on academic performance. Specifically, longitudinal studies could investigate whether sustained motivation and engagement are influenced by a combination of learning styles and teaching strategies over time. Additionally, interdisciplinary and cross-cultural comparisons could further illuminate the global relevance of these findings, offering insights into how learning styles and motivation vary across educational contexts.

A promising avenue for future research is the exploration of AI-driven learning technologies that can adapt teaching methods in real-time based on student performance and engagement. These technologies have the potential to create more personalized and adaptive learning environments, enhancing both engagement and academic performance. Investigating how such innovations can be tailored to different learning styles and motivation levels represents a significant opportunity for improving educational practices in medical education.

### Limitation

Although it does not affect the quantitative statistical analyses, the fact that only 26 first-year students volunteered can be considered a limitation.

This study highlights the complex interplay between learning styles, motivation, and academic performance in medical students. While learning styles alone do not directly predict academic outcomes, they significantly influence student engagement, particularly in hands-on, practical settings. The findings emphasize that intrinsic motivation plays a more crucial role in academic performance than learning style preferences. Medical curricula should therefore prioritize fostering intrinsic motivation and offer diverse, interactive teaching methods that cater to different learning preferences. By integrating personalized feedback, student-centered learning, and mentorship, medical educators can create a more inclusive and effective learning environment.

Future research should focus on the long-term effects of learning styles and motivation on academic performance, with particular attention to emerging technologies like AI-driven adaptive learning tools. These innovations hold the potential to enhance personalized education and student engagement, offering valuable opportunities for improving medical education practices globally. Ultimately, this study underscores the need for a more holistic approach that combines learning styles with motivational strategies to optimize both engagement and academic performance.

## Supplementary Materials

Supplementary Material

## Figures and Tables

**Table 1.** The Frequency Analysis Results of Socio-Demographic Characteristics

		Frequency	%
Year	1st year	26	18.3
	2nd year	54	38.0
	3rd year	62	43.7
Gender	Female	92	64.8
	Male	50	35.2
Type of high school	Science high school (public)	91	64.1
	Science high school (private)	4	2.8
	Private school	6	4.2
	Public high school	38	26.8
	Other	3	2.1
Which educational method do you enjoy the most?	Problem-based learning	18	12.7
	Lectures	13	9.2
	Laboratory skills trainings	34	23.9
	Clinical skills trainings	49	34.5
	Clinic visits	22	15.5
	Other	6	4.2
Which methods do you prefer to study/learn with?	Listening	80	26.0
	Writing	62	20.1
	Reading	87	28.2
	Touching	70	22.7
	Other	9	2.9
Have you received any mentoring/guidance services or training regarding learning or learning methods at any time during your undergraduate education?	Yes	15	10.6
	No	127	89.4

**Table 2.** Relationships Between Learning Styles and Dependent Variables

Variable		Learning Styles*				Total	Pearson Chi-Square (*P*)
		V	A	R/W	K		
Year	Year 1 (n = 26)	3	7	6	10	26	6.828 (.337)
	Year 2 (n = 54)	11	11	8	24	54	
	Year 3 (n = 62)	14	22	9	17	62	
Gender	Female (n = 92)	23	21	11	37	92	10.591 (.014)
	Male (n = 50)	5	19	12	14	50	
Type of high school	Science HS (Public)	18	25	18	30	91	10.572 (.472)**
	Science HS (Private)	1	0	1	2	4	
	Private School	1	3	0	2	6	
	Public HS	8	10	3	17	38	
	Other	0	2	1	0	3	
Which educational method do you enjoy the most?	PBL	2	4	5	7	18	10.348 (.805)**
	Lectures	1	4	3	5	13	
	Laboratory Skills	6	9	7	12	34	
	Clinical Skills	14	12	6	17	49	
	Clinic Visits	4	9	1	8	22	
	Other	1	2	1	2	6	
Have you received any mentoring/guidance services or training regarding learning or learning methods at any time during your undergraduate education?	Mentoring: Yes	3	4	4	4	15	1.707 (.659)**
	Mentoring: No	25	36	19	47	127	

*A, aural; HS, high school; K, kinesthetic; R/W, read/write; V, visual.

**Fisher’s exact test was applied.

**Table 3.** The Themes, Sub-Themes and Some Quotes Revealed from Focus Group Interviews with Students

Theme 1 Learning Motivation	Sub-theme 1.1: The Impact of Teaching Methods	“Seeing the cadaver and receiving practical education in anatomy motivates me more.” FFG “Some lessons feel like storytelling, which lowers my motivation. Practical lessons are more appealing to me.” OFG
	Sub-theme 1.2:The Role of Instructors	“Anecdotes shared by the instructor and connections made to Nobel-winning topics keep me engaged.” BFG “Not stating learning objectives at the beginning makes it harder for me to understand why the lesson is important.” EFG “When teachers read from slides, it discourages me from participating in the lesson.” O2FG
Theme 2 Style for Practical Education	Sub-theme 2.1:Laboratory Lessons	“Peer education in the anatomy lab is much more enjoyable and instructive for me.” CFG “In physiology labs, some lessons are very productive, but in others, we just spend time writing.” E2FG “Working with concrete models in the anatomy lab is the most effective learning method for me.” YFG
	Sub-theme 2.2:Simulations and Practical Training	“Training with people acting as patients makes me feel more prepared for situations I might face in the future.” BFG “Simulated patient training helps us improve our information management.” FFG
Theme 3 Use of Visual and Auditory Materials	Sub-theme 3.1: The Importance of Visual Materials	“Seeing topics like the immune system through animations makes it easier for me to understand.” D2CFG “Using visuals in slides is more effective than just reading text.” E2FG
	Sub-theme 3.2: Use of Written Materials	“Taking notes is the fastest learning method for me; simple slides make it easier for me to learn.” EFG “When there is too much information on the slides, studying becomes harder; they should be simpler and more concise.” BFG “Having the lesson presented in a systematic and sequential way helps me learn the information more effectively.” F2FG
Theme 4 Peer Education	Sub-theme 4.1: Peer-supported Learning	“Learning through peer education is easier because there are no communication barriers, and learning happens faster.” CFG “Working with peers in the anatomy lab helps me understand the subject better.” YFG “PBL sessions and peer education are among the methods that motivate me the most.” E2FG
Theme 5 Challenges in Theoretical Lessons		“Instead of reading from slides, teachers sharing their experiences increases my interest in the lesson.” AOFG “It’s hard to make connections between lessons, which decreases my motivation.” GFG “The systematic and sequential presentation of lessons makes a big difference in how I understand the information.” F2FG
Theme 6 Learning Styles and Individual Differences		“Logic-based lessons motivate me more, but I fall behind in lessons that require memorization.” AEFG “The way teachers explain the lesson is very important for me; the way it is taught is the biggest factor in my motivation.” NFG “Working with computer applications and models makes it easier for me to learn.” BFG

**Table 4.** The Comparison of Educational Methods

**The Revealed Education Method**		**Qualifications**
Lecture hall classes	Strengths	Ability to address large student groups: Lecture hall classes can be attended by large groups, allowing information to be conveyed to many students simultaneously. Use of visual materials: Slides and visuals can capture students’ attention.
	Weaknesses	Low interaction: Students are generally passive listeners, with limited opportunities for questions and discussions. Attention loss: Especially in longer lessons, attention loss and decreased student interest in the lesson are frequently observed.
Multidisciplinary sessions	Strengths	Case-based education: Real cases and a multidisciplinary approach help students relate theoretical knowledge to practical applications by examining topics from different perspectives. Connection between topics: The integration of different disciplines provides a broad perspective for learning.
	Weaknesses	Low participation: There is generally limited interaction and opportunities for questions. While students may engage with the cases, they often remain passive. Attention loss: Long sessions and presentations focused solely on lectures result in students losing attention over time.
Laboratory lessons	Strengths	Hands-on learning: Students actively participate in the learning process through practical activities like microscope examinations and experiments. One-on-one interaction: Strong educator-student interaction; instructors have the opportunity to answer students’ questions directly.
	Weaknesses	Control of the environment: Crowded laboratories can be noisy, and instructors may not be able to give enough attention to all students simultaneously. Difficulty in equal participation: Some students may participate more actively in laboratory activities, while others remain passive.
Problem-based learning (PBL) sessions	Strengths	High interaction: Students actively engage in group discussions and scenario analysis. Student-centered: PBL allows students to manage their own learning processes and discover knowledge. This method encourages critical thinking and research.
	Weaknesses	Lack of materials: In some sessions, the lack of visual and auditory materials may limit student interest. Suitable for smaller groups: It can be difficult to implement with large student groups, as direct interaction is essential in small groups.

**Table 5.** The Themes, Sub-Themes and Some Quotes Revealed From In-depth Interviews with Students

Theme 1 Effectiveness and Contributions of Educational Methods	Sub-theme 1.1: Advantages and Disadvantages of Theoretical Education	“Theoretical lessons are sometimes overloaded, and long slides make the learning process exhausting.” AHKI “Theoretical lessons include useful information, but it’s hard to stay focused in some classes due to low motivation.” AKI
	Sub-theme 1.2:Retention of Practical Education	“Seeing real samples under the microscope in microbiology lab helped reinforce what we learned in theory.” AYI “Working with models in the anatomy lab was very effective; learning by touch makes it more permanent.” ACI
	Sub-theme 1.3: The Positive Impact of Problem-Based Learning (PBL) Sessions	“Doing research within the group during PBL to deeply explore a topic helps reinforce my learning.” AAYI “Solving a topic as a group in PBL is both fun and instructive because we learn new things through research.” ATI
Theme 2 The Effect of Physical Conditions and Resources on Learning	Sub-theme 2.1: Lack of Resources and Material Deficiency	“There were very few models to work with in the anatomy lab. We had to wait our turn, as everyone wanted to use them at the same time.” ACI “Material scarcity and crowding in the lab slow down the learning process.” AHKI
	Sub-theme 2.2:The Effect of Class Size on Learning	“Lecture halls are too crowded; even asking questions is difficult, which makes me passively listen to the lesson.” ATTI “Working in small groups, especially in practical lessons, is more effective because instructors can give more individual attention.” AYI
	Sub-theme 2.3:Time Management and Interaction in Laboratories	“Having to look at the same model multiple times in the lab causes a waste of time. There should have been more models.” ATI “Limited time and materials in lab classes prevent us from asking more questions.” AHKI
Theme 3 Interaction Between Instructors and Students	Sub-theme 3.1:The Role of Instructors	“Professor XXX’s way of speaking and the slides she prepared make the lesson clearer and increase my interest.” AKI “Instructors’ simple explanations and use of visuals make learning easier.” AHKI
	Sub-theme 3.2:Lack of Individual Feedback	“Not receiving one-on-one feedback from instructors in labs made it harder to know whether I was doing things correctly.” ATTI “Getting individual feedback in crowded classes is almost impossible, so we don’t fully know what we are learning in class.” AYI
Theme 4 Student Motivation and Class Participation	Sub-theme 4.1:Compulsory Participation	“I only attend some classes because it’s required. There are very few lessons that genuinely interest me.” AKI “Theoretical lessons are important because they will be useful later, but they can be challenging in terms of motivation.” ACI
	Sub-theme 4.2:Interest in Practical and Professional Training	“Learning how to wear sterile gloves in the basic medical practices class motivated me more.” ATTI “Practical lessons make me feel more like a future doctor, and that increases my motivation.” AAUI
Theme 5 Personal Learning Styles	Sub-theme 5.1: Style for Visual and Practical Learning	“Working with models in the anatomy lab is more memorable than theoretical information.” ACI “Learning with visuals is more effective for me because I understand better what I need to learn in a concrete way.” AYI
	Sub-theme 5.2:The Effect of Individual Study	“Individual study is more productive for me. I struggle to focus during group work.” ATTI “I learn a topic better when studying alone, so I prefer individual study over group work.” AKI
Theme 6 Learning Challenges and Barriers	Sub-theme 6.1: Challenges with Course Content and Memorization	“Some lessons contain too much detail, and memorizing all that information is really difficult.” AHKI “Courses like medical biology, which require a lot of memorization, distance me from the learning process.” AKI
	Sub-theme 6.2:Attention Distraction and Time Management	“The lessons are too long, and I find it difficult to focus. It’s nearly impossible to concentrate for 45 minutes straight.” AAUI “When too much information is given in a lesson, I completely lose focus over time.” AHKI
	Sub-theme 6.3:Exam and Assessment Anxiety	“Constant exams stress me out. I can’t fully focus on the lesson because of exam anxiety.” AYI “The heavy weight of exams in memorization-based lessons makes the learning process more difficult for me.” AAYI

## Data Availability

The data that support the findings of this study are available on request from the corresponding author.
