# Supplementary material for: Exploring the Role of Learning Styles and Motivation in Medical Student Engagement and Academic Performance: A Mixed Methods Study
Source: Eurasian J Med. 2025 Sep 3;57(2):e25862. doi: 10.5152/eurasianjmed.2025.25862 (PMC12557421; doi:10.5152/eurasianjmed.2025.25862)
Supplement: Supplementary Material [file supplementary_material.pdf]

Supplementary Material 1. VARK Questionnaire

How Do I Learn Best?

Choose the answer that best describes your preference. If more than 1 option applies, you may select multiple options. If none of the answers fits your preference, leave the question unanswered.

1. You want to help someone who needs to get to an airport, city center, or train station in your city.
   a. You take them to their destination.
   b. You explain the directions verbally.
   c. You write down the directions for them.
   d. You draw a map or give them one.
2. You are unsure whether a word should be spelled as "anlyarak" or "anlayarak," "nergis" or "nergiz."
   a. You visualize the word in your mind and choose based on how it looks.
   b. You say the words out loud and pick the one that sounds right.
   c. You look it up in a dictionary.
   d. You write both down and select the one that looks correct.
3. You are planning a trip for a group and want to get feedback.
   a. You explain the plan verbally to the group members.
   b. You show them maps or a website with the itinerary.
   c. You give them a printed copy of the route.
   d. You send them the information via phone, fax, or email.
4. You are preparing a special meal for your family.
   a. You make a dish you already know without needing a recipe.
   b. You ask for suggestions from friends.
   c. You look through a cookbook for ideas from the pictures.
   d. You follow a trusted recipe from a book.
5. A group of tourists wants to learn about the local parks and wildlife in your area.
   a. You talk to them about the parks or arrange a talk.
   b. You show them pictures or images from the internet.
   c. You take them to a park or wildlife area and give them a tour.
   d. You give them a book or brochure about the parks and wildlife.
6. You are deciding which digital camera or mobile phone to buy. Besides the price, what most influences your decision?
   a. Trying it out to see how it feels.
   b. Reading detailed information about its features.
   c. Its modern design and look.
   d. The salesperson's explanation of the features.
7. Think of a time when you learned how to do something new. What helped you learn best?
   a. Watching it being done.
   b. Listening to someone explain it and asking questions.
   c. Diagrams, shapes, and visual cues.
   d. A manual or written instructions.
8. You have an issue with your knee. Which of the following would you prefer the doctor to do?
   a. Provide a source (e.g., website) to read about the issue.
   b. Show a plastic model of the knee to explain the problem.
   c. Verbally explain the issue.
   d. Use a diagram to show the issue.
9. You want to learn how to use a new computer program, skill, or game.
   a. You read the written instructions or manual provided.
   b. You talk to someone who knows how to use it.
   c. You try using the controls or keyboard yourself.
   d. You follow the visual diagrams in the booklet.
10. What do you like most about your favorite websites?
   a. Features that you can click, change, or try out.
   b. Interesting design and visual elements.
   c. Engaging written descriptions and lists.
   d. Audio channels like music, radio shows, or talks.
11. When choosing a science fiction book, what influences your decision the most (aside from price)?
   a. Its attractive appearance.
   b. Reading a few chapters quickly.
   c. A recommendation from a friend.
   d. Realistic stories and examples that you can relate to.

12. You are learning to use your new digital camera using a booklet, CD, or website. What do you prefer the resource to include?
   a. An opportunity to ask questions or talk about the camera's features.
   b. Clear written instructions with a checklist of steps.
   c. Diagrams showing each part of the camera.
   d. Examples of good and bad photos, and how to improve them.
13. Which type of teacher do you prefer?
   a. One who uses demonstrations, models, or practical activities.
   b. One who uses question-and-answer sessions, talks, group discussions, or guest speakers.
   c. One who gives written notes, books, or reading materials.
   d. One who uses diagrams, charts, or graphs.
14. You have finished a competition or exam and want feedback on your performance. Which form of feedback do you prefer?
   a. Examples of what you did.
   b. A written explanation of your results.
   c. A detailed conversation about your performance.
   d. Graphs showing your performance against benchmarks.
15. You are choosing food at a restaurant or cafe. How do you decide?
   a. By ordering the same dish you have had before.
   b. By asking the waiter or getting recommendations from friends.
   c. By reading the menu descriptions.
   d. By looking at what others are eating or at pictures of the dishes.
16. You have to give an important speech at a conference or special event. How do you prepare?
   a. You use diagrams and charts to clarify key points.
   b. You write down key words and practice the speech several times.
   c. You write out the full speech and read through it multiple times.
   d. You collect lots of examples and stories to make the speech more realistic and relatable.

The VARK Questionnaire Scoring Chart

Use the following scoring chart to find the VARK category that each of your answers corresponds to. Circle the letters that correspond to your answers

e.g. If you answered b and c for question 3, circle V and R in the question 3 row.

Question	a category	b category	c category	d category
3	K	V	R	A

Scoring Chart

Question	**a** category	**b** category	**c** category	**d** category
**1**	K	A	R	V
**2**	V	A	R	K
**3**	K	V	R	A
**4**	K	A	V	R
**5**	A	V	K	R
**6**	K	R	V	A
**7**	K	A	V	R
**8**	R	K	A	V
**9**	R	A	K	V
**10**	K	V	R	A
**11**	V	R	A	K
**12**	A	R	V	K
**13**	K	A	R	V
**14**	K	R	A	V
**15**	K	A	R	V
**16**	V	A	R	K

Calculating your scores
Count the number of each of the VARK letters you have circled to get your score for each VARK category.

Total number of **V**s circled =

Total number of **A**s circled =

Total number of **R**s circled =

Total number of **K**s circled =

Supplementary Material 2. Socio-demographic Information Form

1. What is your year of study?
   - 1st year
   - 2nd year
   - 3rd year
2. What is your gender?
   - Female
   - Male
3. What type of high school did you attend?
   - Science High School (Public)
   - Science High School (Private)
   - Private School
   - Public High School
   - Other
4. Which educational method do you enjoy the most?
   - Problem-Based Learning (PBL)
   - Instructor-led presentations (including lecture hall classes)
   - Laboratory lessons
   - Clinical skills training
   - Field visits (e.g., outpatient clinics, Family Health Centers, etc.)
   - Other: _________
5. Have you received any mentoring/guidance services or training related to learning or learning methods during your undergraduate education?
   - Yes
   - No

Supplementary Material 3. Educational Activity Observation Form

Name of the Educational Activity:

Date:

Name(s) of the Instructor(s):

Location of the Educational Activity:
- Online
- Basic Science Laboratories
- Lecture Hall
- Student-Centered Education Activity Room
- Objective Structured Clinical Examination (OSCE) Center
- Other: ______________________________

Method of the Educational Activity:
- Problem-Based Learning (PBL)
- Laboratory Educational Activity
- Feedback Presentation
- Multidisciplinary Session
- Clinical Skills Training
- Other: ______________________________

Number of students at the beginning of the educational activity (within the first 10 minutes):

……………………………………………………………………………………………..

Number and duration of activities/behaviors performed by the instructor to prepare, motivate, or capture students' attention at the beginning of the educational activity:

………………………………………………………………………………………………

Students' attitudes/behaviors toward the instructor's attempts to prepare, motivate, or capture their attention at the beginning of the educational activity:

……………………………………………………………………………………………..

Number of students who left the educational activity before its end and at what time:
- In the first third of the session: ________
- In the middle third of the session: ________
- In the final third of the session: ________

Percentage of students disengaged from the educational activity (e.g., sleeping, stretching, distracted, talking to each other):
Note the observations for each third of the session.
- In the first third of the session: ________
- In the middle third of the session: ________
- In the final third of the session: ________

Noise level during the educational activity:
- None
- Mild
- Acceptable
- Distracting

Number of students participating/asking questions during the educational activity:
- Number of students: ________

Time of the first question asked: _______ minutes
Number of questions asked: _______ questions

Applications/interactive activities used during the educational activity (those actively involving students):
- First application time: ______ minutes
- Number of applications: ______
- Percentage of total duration: ______
- Content: ____________________________

(For lecture halls) Seating arrangement of students during the educational activity:
- At the front
- At the back
- Homogeneous
- Scattered

Students' attention during the educational activity:
- Fully engaged
- Mostly engaged
- Partly engaged
- Distracted

Number of students approaching the instructor to ask questions during or after the educational activity:

……………………………………………………………………………………………..

Observations of professional attitudes during the educational activity:
- Teamwork
- Communication Skills
- Taking Responsibility
- Researcher
- Leadership

Students' responses to visual methods or educational materials during the educational activity:
- Paying close attention
- Increased motivation
- No change
- Other: ____________________________

Students' responses to auditory educational materials during the educational activity:
- Paying close attention
- Increased motivation
- No change
- Other: ____________________________

Students' responses to tactile education methods (e.g., standardized patients, models) during the educational activity:
- Paying close attention
- Increased motivation
- No change
- Other: ____________________________
